# The Quality of Life of Coronavirus Disease Survivors Living in Rural and Urban Area of Riau Province, Indonesia

**DOI:** 10.3390/idr14010005

**Published:** 2022-01-07

**Authors:** Suyanto Suyanto, Shashi Kandel, Rahmat Azhari Kemal, Arfianti Arfianti

**Affiliations:** 1 Faculty of Medicine, Universitas Riau, Kota Pekanbaru 28133, Indonesia; rahmat.azharikemal@lecturer.unri.ac.id (R.A.K.); evi_anti@yahoo.com (A.A.); 2 Epidemiology and Disease Control Division, Ministry of Health and Population Nepal, Kathmandu 44600, Nepal; sawsee1985@gmail.com

**Keywords:** quality of life, COVID-19, SGRQ score

## Abstract

This study assesses the status of health-related quality of life (HRQOL) among coronavirus survivors living in rural and urban districts in Riau province, Indonesia. The cross-sectional study was conducted among 468 and 285 Coronavirus disease (COVID-19) survivors living in rural and urban areas, respectively in August 2021. The St. George Respiratory Questionnaire (SGRQ) was used to measure the HRQOL of COVID-19 survivors. A higher total score domain corresponds to worse quality of life status. Quantile regression with the respect to 50th percentile found a significant association for the factors living in rural areas, being female, having comorbidities, and being hospitalized during treatment, with total score of 4.77, 2.43, 7.22, and 21.27 higher than in their contra parts, respectively. Moreover, having received full vaccination had the score 3.96 in total score. The HRQOL of COVID-19 survivors living in rural areas was significantly lower than in urban areas. Factors such as living in rural areas, female sex, having comorbidities, and history of symptomatic COVID-19 infection were identified as significant predictors for lower quality of life. Meanwhile, having full vaccination is a significant predictor for a better quality of life. The results of this study can provide the targeted recommendations for improvement of HRQOL of COVID-19 survivors.

## 1. Introduction

COVID-19 is inevitably one of the worst pandemics in modern human history felt by various levels of worldwide society, which affects wellbeing not only physically, but also psychologically and socially [1,2,3]. COVID-19 was first reported in China in December 2019. By August 2021, it had reached more than 216 million confirmed cases and has led to 4.5 million deaths in 221 countries [4]. Many countries are still struggling to contain the transmission of COVID-19, which resulted in severe pressure on health care systems and financial burdens.

Most COVID-19 cases recovered completely, but some of them could experience the symptoms for months or even years. Some studies reported that survivors still endure persistence of symptoms and develop psychiatric and mental health problems in the longer term [2,5,6,7,8]. These post-acute sequelae of COVID-19 (PASC) or “long-COVID” symptoms can be mild, without interfering with daily activities, but it can also be severe, disturbing daily physical activities and psychosocial life (worry, lack of energy, depression, and sometimes stigmatization). Consequently, it impacts the quality of life of coronavirus survivors. Health-related quality of life is a subjective experience of patients regarding the impact of the disease on disruption of daily activities and emotional disturbances [9,10]. Older people, people with pre-existing medical conditions (such as diabetes, high blood pressure, and heart disease), and people who were hospitalized during treatment are usually more susceptible of having poor quality of life several months after recovering from COVID-19 infection [2,8]. 

Indonesia recorded more than 4 million cases of COVID-19, making it one of the worst-affected countries in the world [11,12]. Of these, 3.8 million cases have been declared cured and about 130,000 people died [13]. In order to suppress the increasing number of cases, the Indonesian government emphasizes the importance of health behaviors, namely wearing masks, washing hands, maintaining distance, avoiding crowds, and limiting mobility. In addition, the administration of the COVID-19 vaccine began in early January 2021. Given a large number of daily new confirmed cases of COVID-19, there are still not many programs to pay attention to COVID-19 survivors.

Studies evaluating the quality of life of COVID-19 survivors in developing countries are still scarce. Moreover, there is a paucity of information on which factors have any effects on the quality of life. The individual perception of quality of life is related in the context of the culture, gender, level of education, and value system in which they live. From preliminary studies (personal unpublished data), it is known that COVID-19 survivors often consider themselves cured so that they do not continue treatment from health services even though some of them experience PASC symptoms and need to consult a doctor. Our study aimed to investigate the survivor’s quality of life six months after being discharged from COVID-19 treatment and to determine factors associated with quality of life. This study result would enable the healthcare professionals and policy makers to have a comprehensive understanding of the quality of life among COVID-19 survivors.

## 2. Materials and Methods

### 2.1. Study Design and Setting

**Figure 1:**
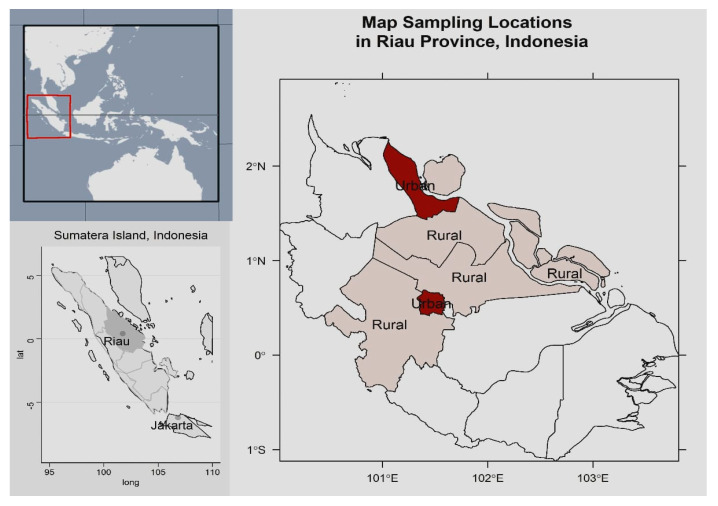
Geographical Location of Riau Province, Indonesia.

The study was conducted in Riau Province, located on Sumatera Island, Indonesia. This province has two urban and ten rural districts (Figure 1). Of these districts, two urban and four rural districts were chosen based on accessibility and number of confirmed cases between December 2020 to February 2021. The cross-sectional survey using the St. George Respiratory Questionnaire (SGRQ) questionnaire was carried out to obtain Health Related Quality of Life (HRQOL) data within August 2021. The study protocol was approved by the Research Ethics Committee of Faculty of Medicine, Riau University. 

### 2.2. Study Population

The required sample size was calculated based on detecting a minimum clinically important difference between the means of the two groups [14]. The minimum sample size is 99 per group, assuming equal group sizes, for detecting a true difference in means of 4 points and a standard deviation of 10 points between two groups. Then, the participants were extracted from the population frame who met the inclusion criteria, including the survivors who have a valid phone number, live permanently in that district, and were aged 18 years or above. Survivors who were unfit or pregnant at the time of the study were excluded. There was no restriction on the total number of participants, however, a minimum target of 400 participants for urban and 400 participants for the rural district was desired.

### 2.3. Data Collection

Eligible participants were contacted by phone and the research was explained by research assistants. Research assistants ensured that the respondents understood the aim of the research well and written informed consent was obtained. Then, the research assistants interviewed participants by phone and filled out the questions. This questionnaire contains:

1. Participant characteristics: Age, sex, education level, income group, comorbidities, vaccination status, and disease severity. Age was categorized into young (≤51 years old) and elder (>51 years old). Educational level was defined by highest educational level and categorized into low (primary and secondary education) and high (university education). Income was categorized into having daily permanent or non-permanent income. Comorbidities included tuberculosis, diabetes, hypertension, asthma, or chronic obstructive pulmonary disease, and further categorized into having comorbidity and no having comorbidity. The vaccination status was categorized into fully, partially, and non-vaccinated. Disease severity was classified as moderate/severe, mild, and asymptomatic.
2. Health Related Quality of Life: The St. George’s Respiratory Questionnaire (SGRQ) Indonesia language, which is a specific instrument to assess quality of life for several chronic respiratory disorders, was used. This SGRQ test assessed four domains: symptoms, activity, impact, and total scores. Symptom domain assesses patient perception in the past of their recent respiratory problems related with cough, difficulty to breathe, or chest pain. Activity domain assesses patient disturbances to daily physical activity on current date. Impact domain assesses patient disturbances of psycho-social function on current date. The SGRQ score of symptom, activity and impact domains was obtained with the scoring calculator (Microsoft Excel-based) provided with the instrument score and a total score, which reflect the proxy for quality of life, can be generated. This scaled from zero (optimal health) to one hundred (worst health). A higher score corresponds to worse health-related quality of life [15].

### 2.4. Statistical Analysis

Data were analyzed using R statistical software [16]. The descriptive analysis was summarized as mean with standard deviation or median with interquartile range for continuous variables and percentages for categorical variables. Significant tests with a *p*-value of <0.05 using the Kruskal–Wallis test were considered statistically significant and used for describing the comparisons between predictors and the outcome (SGRQ). 

**Figure 2:**
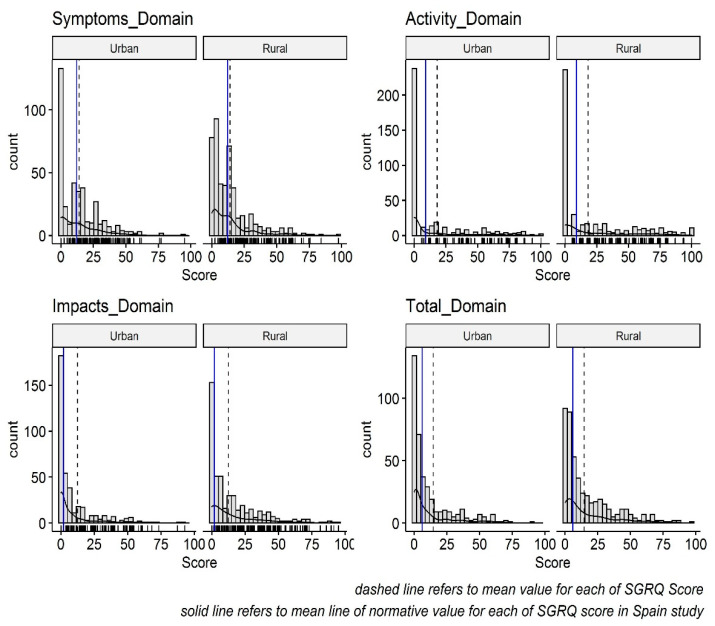
Frequency Distribution of SGRQ score domain between rural and urban region.

As the SGRQ scores were distributed non-normally on all domains of SGRQ for both participants in rural and urban groups (Figure 2), quantile regression models were constructed to determine a relationship between the 50th percentiles of a continuous SGRQ score and a set of predictors. Univariate quantile regression was used initially to determine the candidate of independent predictors (Survivor characteristics) associated with the dependent variable (SGRQ score). Variables with *p*-values < 0.2 were selected in the multivariate model along with the survivor living region, the main hypothesis variable. 

## 3. Results

### 3.1. Respondent Characteristics 

A total of 853 participants (45.1% living in urban areas) completed the survey and their data were included in the analysis. The data shown in Table 1 describe the comparison values of the participant characteristics by region residence in absolute numbers as well as percentages of the values. There were no significant differences in the distribution of age, sex, education level, and comorbidities between rural and urban regions. Participants from the rural region had a significantly higher proportion of permanent income, complete vaccination, and moderate/severe COVID-19 than those from the urban region. 

### 3.2. Comparison Domain of SGRQ Score between Participant Characteristics

The difference of each SGRQ score stratified by the participant characteristics is presented in Table 2. Symptoms’ scores were not significantly different between the two regions of residence groups. Conversely, activity, impact, and total scores of the urban group were significantly lower than those of the rural group. Older survivors had a higher score in each domain of SGRQ, i.e., symptoms, activity, and impact. Hence, leading to a higher “total score” compared to the younger survivors. The symptoms’ score was significantly higher on survivors who have comorbidities and had moderate/severe clinical manifestation. Similarly, being female, having comorbidities, and hospitalization had significantly higher scores in the “activity “and “impact” domain forging a higher “total” score. Our study also found that fully vaccinated survivors had lower activity, impact, and total scores compared to unvaccinated and partially vaccinated survivors.

The value of SGRQ score domains indicated median (inter-quartile range), unless otherwise stated.

### 3.3. Factors Associated with SGRQ Score 

Table 3 presented the association between the predictors and SGRQ score for each domain by quantile regression in respect of the 50th percentile. Residing in a rural region was associated with significantly increased symptoms, activity, impact, and total scores. Being female was significantly associated with a higher score of activity as well as total scores. Symptomatic (mild and moderate/severe) COVID-19 and existing health condition history were associated with significantly increased symptom, activity, impact, and total scores. Participants receiving full and partial vaccination had significantly higher scores on activity, impact, and total domains. Considering the “Total Score” as a proxy of the coronavirus survivor’s quality of life, factors such as living in rural areas, female sex, having comorbidities, and history of symptomatic COVID-19 infection were identified as significant predictors for lower quality of life. Meanwhile, having full vaccination is a significant predictor for a better quality of life. 

## 4. Discussion

This study evaluates the quality of life among COVID-19 survivors 6 months after hospital discharge. A comparison between survivors living in the rural region to urban region was performed. Findings show survivors living in the rural region reported a subsequently lower quality of life than in the urban region. Furthermore, being female, having comorbidity, and hospitalization were found to be significantly associated with worse quality of life, while having full or partial vaccination post-COVID-19 was found to be significantly associated with better quality of life. Though there was limited evidence, older survivors indicated having higher symptoms, activity, impact, and total score.

Lower HRQoL was observed among survivors living in rural regions. Both urban and rural regions in Indonesia have public health centers (*pusat kesehatan masyarakat*, *puskesmas*) and local general hospitals providing free COVID-19 treatment. However, there might be differences in facilities provided that could affect the HRQoL. Additionally, differences in cultural characteristics and geographic factors between urban and rural regions might also affect health-seeking behaviors and access to healthcare facilities. This might result in unmet medical care need for those who live in rural areas [17], as delay in reaching the healthcare facility and receiving the medical care during initial phase of the disease might have played a role in a worse quality of life.

The findings are consistent with previous findings indicating HRQOL in COVID-19 survivors remained lower [1,2,5,6,8,11,18]. Most COVID-19 survivors experience a decrease in their quality of life when compared to their quality of life before suffering from COVID-19. However, there are not many studies conducted assessing the health-related quality of life using SGRQ instruments in the context of COVID-19 survivors. We do not have the normative data for the general Indonesian population, however, the average SGRQ score in our study participants was higher than on those reported for the mean normative value in a Spanish study [19], which reported an average symptoms score of 12, activity score of 9, impact score of 2, and total score of 6. The reason for the higher symptoms score may be related to persisting residual respiratory symptoms due to the pulmonary function impairment or other underlying co-morbid conditions that affect patients for a longer term. In addition, some survivors had persisting arthralgia or myalgia symptoms and electrolyte imbalance that may limit physical activity and contribute to higher activity score. Furthermore, the higher activity score interferes with psychosocial life, as shown as the higher impacts score.

In accordance with the results of other studies, being female [18], being hospitalized [20,21], and having a comorbidity [22] were significantly associated with worse quality of life. Women tend to report poorer health as they are more aware of health, but this perception may differ among different cultures. Furthermore, women commonly perceive significantly higher levels of post-traumatic stress than men, which may eventually have a greater negative impact on their quality of life. A study from China mentioned the female sex as a risk factor for low quality of life (especially mental health) after COVID-19 infection [23]. In addition, differences in coping strategies between males and females may explain the gender differences in addressing such health issues [24]. Hence, women might have experienced or witnessed poor quality of life compared to males and priority should be given to such a vulnerable population even after hospital discharge.

In this study, comorbidity was significantly associated with poorer quality of life. Similar findings, i.e., comorbidities such as hypertension, chronic pulmonary disorders, any chronic disorders, were identified as a culprit for persistent post COVID-19 symptoms [25] that may hinder quality of life. There is the possibility of some patients with comorbidities to develop bronchial and parenchyma sequelae which is attributable to COVID-19 severity. Providing more attention towards those groups, for example in the form of regular medical evaluation as well as family support, may benefit their quality of life. 

All of moderate/severe survivors in our study were hospitalized during their treatment. As shown in Table 3, the survivor who got hospitalization was likely to show worse quality of life. An analogous finding was mentioned in a study from Israel; patients requiring hospitalization with COVID-19 reported poor quality of life and needs prolonged medical follow up [26]. In addition to this, a Brazilian study highlighted that an ICU admission was an independent predictor of worsening health related quality of life [27]. 

Our study found that being fully vaccinated contributes significantly to better HRQOL. Although it cannot fully protect a person from COVID-19 infection, vaccination can reduce the possibility of severe symptoms and complications. There are several hypotheses regarding the mechanism of post-acute sequelae of COVID-19 (PASC), one of them is viral persistence. Therefore, if viral persistence is observed, vaccination might be able to induce cellular and humoral responses to eliminate the viral reservoir. There is currently a study investigating the mechanism of how vaccination might affect PASC symptoms. [28] During our study time in August 2021, the most commonly used vaccine in Indonesia and in Riau was inactivated vaccine, CoronaVac (Sinovac, China). Adenoviral-vectored vaccine, Vaxzevria (Astrazeneca), has been authorized for emergency use in Indonesia since March, however, its utilization prior to July 2021 was initially low. Therefore, although we did not ask the vaccine type received by respondents, we assumed that most of our respondents received CoronaVac vaccine.

Most studies report that age is associated with quality of life [5,11,21,22,29]. However, our study failed to determine if age is associated with quality of life. This observation might be driven by the fact that the majority of our respondents were relatively young. However, though there is limited evidence, the older group had a higher score in all of the SGRQ domains. 

Several limitations need to be noted. Subject recruitment was influenced by the data availability from District Health Offices as well as the willingness of participants to be involved. Respondents who personally know the interviewers might be more inclined to respond to the invitation. Recruiting the subjects, especially those living in an urban region, was more challenging compared to a rural region. More people living in the rural region responded to our research invitation. Additionally, the subjects who were experiencing symptoms might be more inclined to participate than those who had no symptoms, and therefore the results should be interpreted carefully.

SGRQ reflects perceptions in the current time, whereas question asked [29]. The answer also depends on the respondent’s awareness and personality background that can be associated with their perceived HRQOL. In addition, as SGRQ consists of a lot of questions and may consume time, there were about 10% of participants who failed to complete all of the questions. Comorbidity status of the study subjects needs to be further investigated as we did not perform medical examinations such as pulmonary function tests, chest radiology, or blood laboratory to confirm the underlying diseases that may affect HRQOL. We also did not examine other risk factors of the study participants, for example smoking history, personal hygiene, or nutritional status.

Finally, due to the diverse culture and uneven healthcare facilities across Indonesia, we did not aim to represent the whole Indonesian population. However, our result might represent areas that have similar characteristics to Riau province such as other provinces in Sumatera Island.

## 5. Conclusions

COVID-19 survivors may suffer PASC that affects their quality of life as detected by higher SGRQ scores. Compared with the people who survived COVID-19 and live in urban areas, those who survived COVID-19 and live in rural areas had a lower quality of life. This finding reveals the urgency of identifying and improving the quality of life as an important component in PASC or “Long COVID” management. We propose a periodical post-COVID-19 monitoring and evaluation program for the survivors. The concern should be given to survivors in rural areas, symptomatic survivors, and survivors with comorbidities. Furthermore, as we found that vaccinated survivors had higher quality of life, vaccinating the survivors against COVID-19 remains highly recommended. 

## Figures and Tables

**Table 1:** Characteristics of the study participants.

Characteristics	Rural Region (N = 468)	Urban Region (N = 385)	*p* Value
Age			0.10
Median (IQR)-year	31 (25–41)	29 (23–42)	
Age group			0.60
>51 years (>90% percentile)	36 (9.4)	50 (10.7)	
≤51 years (≤90% percentile)	349 (90.6)	418 (89.3)	
Sex group			0.18
Female	278 (59.4)	247 (64.2)	
Male	190 (40.6)	138 (35.8)	
Education level group			0.28
Average education	165 (42.9)	219 (46.8)	
Higher education	220 (57.1)	249 (53.2)	
Income group			<0.01
Non-permanent income	169 (36.1)	174 (45.2)	
Permanent income	299 (63.9)	211 (54.8)	
Having comorbidity			0.28
Yes	43 (9.2)	45 (11.7)	
No	425 (90.8)	340 (88.3)	
Vaccination status			<0.01
Fully vaccinated	293 (62.6)	186 (48.3)	
Partially vaccinated	46 (9.8)	48 (12.5)	
Unvaccinated	129(27.6)	151 (39.2)	
Disease severity			<0.01
Moderate/Severe	57 (12.2)	27 (7)	
Mild	215 (45.9)	250 (64.9)	
Asymptomatic	196 (41.9)	108 (28.1)	

The value indicated absolute number (percentage) unless otherwise stated. IQR (interquartile range).

**Table 2:** Distribution of SGRQ domain scores for COVID-19 survivors in the study population.

Characteristics	N	Symptoms Score	Activity Score	Impacts Score	Total Score
Overall					
Mean (SD)		13.9 (15.7)	18.1 (27.3)	12.5 (17.7)	14.5 (18.5)
Median (IQR)		9.7 (2.6–18.5)	0 (0–29.6)	4.6 (0–16.3)	6.5 (1.5–21.6)
Regions of residence					
Rural	468	9.7 (2.6–18.2)	0 (0–35.5) *	9.0 (0–22.9) *	8.4 (2.3–25.4) *
Urban	385	9.7 (0–21.7)	0 (0–18.5)	3.8 (0–11.6)	4.3 (0.7–14.0)
Age group					
>51 years	86	12.3 (4.7–19.6)	6.0 (0–43.2)	7.8 (0–19.5)	9.1 (2.2–27.0)
≤51 years	767	9.7 (0–18.3)	0 (0–25.9)	4.2 (0–16.2)	6.2 (1.5–20.1)
Sex group					
Female	525	9.7 (0–18.5)	5.8(0–35.2) *	7.6 (0–17.7) *	7.9 (2.1–24.5) *
Male	328	9.8 (2.6–18.3)	0 (0–12.3)	2.0 (0–15.8)	4.0 (0.7–16.0)
Education level group					
Average education	384	9.7 (2.6–18.3)	0 (0–35.1)	4.2 (0–19.4)	6.4 (1.5–25)
Higher education	469	10.5 (2.3–18.4)	0 (25.2)	4.6 (0–15/6)	6.5 (1.9–19.8)
Income group					
Non-permanent income	343	9.7 (2.6–19.6)	6.0 (0–31.7)	7.6 (0–21.7)	7.8 (1.6–25)
Permanent income	510	9.8 (2.3–18.3)	0 (0–25.1)	4.0 (0–15.6)	5.8 (1.5–19.7)
Having comorbidity					
Yes	88	18.3 (12.2–35.5) *	33.2 (0–67.1) *	11.6 (0–37.7) *	22.2 (4.1–49.5) *
No	765	9.7 (0–17.0)	0 (0–24.1)	4.1(0–15.6)	5.9 (1.5–18.3)
Vaccination Status					
Fully Vaccinated	479	9.7 (2.4–17.7)	0 (0–24.6) *	4.0 (0–15.2) *	4.5 (0.8–17.7)*
Partially Vaccinated	94	10.5 (0–19.6)	0 (0–18.5)	4.0 (0–11.7)	5.5 (2.1–13.7)
Unvaccinated	280	10.5 (2.6–21.3)	11.2 (0–41.6)	8.0 (0–27.5)	9.6 (2.4–27.8)
Disease severity					
Moderate/Severe	84	27.6 (15.5–38.1) *	36.4 (0–68.3) *	18.8 (9.6–48.6) *	26.6 (12.5–52.4) *
Mild	465	12.3 (4.7–22)	5.8 (0–35.6)	6 (0–21.6)	8 (2.1–24.4)
Asymptomatic	304	2.6 (0–12.3)	0 (0–6)	0 (0–8.1)	2.3 (0.4–8.5)

* Significant difference median of score between characteristics groups.

**Table 3:** Coefficient of Quintile regression with 95% confidence interval to predict the median of SGRQ domain score from the predictor exposure.

Predictors	Levels	Symptom Domain	Activity Domain	Impacts Domain	Total Domain
Region of residence	Rural	2.56 *	2.98	3.99 *	4.77 *
		(2.56–2.56)	(0.25–1.46)	(3.43–5.87)	(3.06–5.54)
	Urban	0	0	0	0
Sex group	Female		2.98	3.82	2.43 *
			(0.17–3.97)	(0.16–4.22)	(1.27–3.86)
	Male		0	0	0
Having comorbidity	Yes	8.74 *	15.84 *	4.40	7.22 *
		(4.23–14.65)	(3.73–30.20)	(−0.07–9.83)	(3.12–17.57)
	No	0	0	0	0
Vaccination status	Fully vaccinated		−2.98 *	−3.99 *	−3.96 *
			(−8.68–1.61)	(−5.90–3.15)	(−5.87–2.88)
	Partially vaccinated		−2.98 *	−3.84 *	−3.00
			(−7.86–2.83)	(−4.32–0.25)	(−5.15–0.61)
	Unvaccinated		0	0	0
Disease severity	Moderate/Severe	19.45 *	32.62 *	15.00 *	21.27 *
		(15.65–29.09)	(22.21–38.95)	(10.51–19.42)	(16.22–24.51)
	Mild	9.71 *	2.09	3.97 *	5.18 *
		(9.71–9.71)	(0.51–4.30)	(2.99–4.31)	(4.74–6.43)
	Asymptomatic	0	0	0	0

Values in brackets denote 95% confidence interval. The * indicates significant difference.

## Data Availability

All the primary data and materials involved in this paper are from the primary data source. If readers need more information about data and materials, please contact author for data requests.
